# Thermomechanical Peridynamic Modeling for Ductile Fracture

**DOI:** 10.3390/ma16114074

**Published:** 2023-05-30

**Authors:** Shankun Liu, Fei Han, Xiaoliang Deng, Ye Lin

**Affiliations:** 1 State Key Laboratory of Structural Analysis for Industrial Equipment, Department of Engineering Mechanics, International Research Center for Computational Mechanics, Dalian University of Technology, Dalian 116023, China; shankunliu@mail.dlut.edu.cn; 2 National Key Laboratory of Shock Wave and Detonation Physics, Institute of Fluid Physics, China Academy of Engineering Physics, Mianyang 621999, China; xiaoliangdeng@163.com; 3 Beijing Electro-Mechanical Engineering Institute, Beijing 100074, China; linye1615@sina.com

**Keywords:** peridynamics, thermoelastic coupling, plastic deformation, fracture simulation

## Abstract

In this paper, we propose a modeling method based on peridynamics for ductile fracture at high temperatures. We use a thermoelastic coupling model combining peridynamics and classical continuum mechanics to limit peridynamics calculations to the failure region of a given structure, thereby reducing computational costs. Additionally, we develop a plastic constitutive model of peridynamic bonds to capture the process of ductile fracture in the structure. Furthermore, we introduce an iterative algorithm for ductile-fracture calculations. We present several numerical examples illustrating the performance of our approach. More specifically, we simulated the fracture processes of a superalloy structure in 800 ℃ and 900 ℃ environments and compared the results with experimental data. Our comparisons show that the crack modes captured by the proposed model are similar to the experimental observations, verfying the validity of the proposed model.

## 1. Introduction

Many ductile materials, such as superalloys, are regularly subjected to high temperatures for long periods of time, resulting in irreversible plastic deformation and even ductile fracture. Although several numerical studies on the failure of ductile materials have been conducted [1,2,3], the methods proposed therein are not suitable for predicting crack initiation and propagation processes. This is because that the constitutive models used and the corresponding numerical simulations were based on classical continuum mechanics (CCM). Continuity assumption is one of the basic assumptions of CCM, which requires that the field variables are continuous functions of space and time. The stress divergence term in the equilibrium equation described by CCM requires the second derivative of displacement. However, in the fracture problem, the field variables such as displacement at the crack tip are discontinuous. Therefore, the displacement function at the crack tip is non-differentiable when CCM is used to deal with the fracture problem, that is, the singularity problem appears. To avoid the difficulties repeatedly encountered by CCM, a nonlocal solid mechanics theory called peridynamics (PD) was proposed by Silling in 2000 [4]. In recent years, the advantages of metal fracture modeling and numerical simulations based on PD are gradually becoming apparent [5,6].

PD theroy assumes that interactions still exist between non-contact material points separated by a finite distance. It describes the mechanical behaviors of materials by solving integral equations of nonlocal effects in space, avoiding the singularity that occurs when CCM faces the fracture problem [7,8]. Therefore, PD models are better suited for simulating fracture processed and do not require complex crack propagation criteria [9].

However, nonlocal integration substantially increases calculation costs, and traditional traction boundary conditions cannot be applied directly in PD models. An effective way to solve this problem is to couple PD with CCM [10,11,12,13,14]. Lubineau et al. proposed the Morphing method to couple PD with CCM based on the principle of energy density balance [15], and Azdoud et al. used this method to study quasi-static fracture problems [16]. Yang et al. proposed a coupling model of CCM and ordinary state-based PD to predict brittle fracture under quasi-static and dynamic loading [17]. Shen et al. presented a hybrid dual-variable-horizon PD/CCM modeling approach and a strength-induced adaptive coupling algorithm to simulate brittle fractures in porous materials [18]. Anicode et al. presented the coupling of dual horizon bond-based, ordinary state-based and non-ordinary state-based PD with traditional finite elements in ANSYS [19].

The above PD-CCM coupling models are used to simulate the brittle fracture of elastic materials. However, PD can be used to simulate not only the behavior of linear elastic materials but also the plastic deformation of materials. Macek and Silling proposed a perfectly elastoplastic bond constitutive model by permitting the permanent deformation of individual bonds [20]. Madenci and Oterkus proposed an ordinary state-based PD model for plastic deformation based on von Mises yield criteria [21]. Liu et al. proposed an ordinary state-based PD model for the nonlinear hardening of plastic materials [22]. Furthermore, many scholars have applied the PD-CCM coupling modeling method to simulate ductile fracture. Tong et al. proposed an adaptive coupling method for the combination of the state-based PD and CCM to simulate the progressive failure process in cohesive materials [23]. Liu et al. proposed an element-based PD and CCM coupling model to solve the elastic-plastic deformation and failure problems [24]. Alebrahim et al. proposed a PD-CCM model to simulate cohesive crack growth in quasi-brittle materials [25]. Yang et al. proposed a dynamic PD-CCM coupled model without an overlapped zone for progressive damage analysis [26]. Although great progress has been made in the PD-CCM coupled modeling of ductile fracture, the above models do not consider the effects of temperature. Both thermal and plastic deformations should be considered in simulations of the plastic fracture of certain materials, such as superalloys. In the thermomechanical modeling of PD, Liu et al. presented a thermomechanical ordinary state-based PD model to investigate the fracture mechanisms in nuclear fuel pellets [27]. Zhang et al. proposed a bond-based PD, fully coupled, thermomechanical model for simulation of the fracture in quasi-brittle materials [28]. Song et al. adopted PD to develop an inhomogeneous sea ice model and applied it to simulate crack propagation in a thermomechanical field [29]. Li proposed a weak form of bond-associated PD model for thermomechanical analysis of orthotropic structures [30]. These thermomechanical simulations were modeled only using PD. The thermomechanical PD-CCM concurrent coupling model is rare at present.

Therefore, in this study, we develop a thermomechanical PD-CCM coupled model and a plastic bond constitutive model to simulate the fracture of ductile material at high temperatures. We use the Morphing method to establish a thermoelastic model that couples PD with CCM to limit the plastic PD model to the fracture region, thereby reducing computational costs. In previous studies, the application of morphing coupling method to fracture simulation under elastic stress field [16,31,32] and thermoelastic stress field [33] has been comprehensively discussed. In this paper, the morphing coupling method is further extended to plastic fracture simulations.

The remainder of this paper is organized as follows. Section 2 proposes a new bond-based PD constitutive model that considers plasticity and thermal expansion in bond deformation. Section 3 derives a thermoelastic coupling model between PD and CCM using the Morphing method. In Section 4, we introduce an iterative algorithm for plastic-fracture calculations. In Section 5, we present numerical results for fracture processes in a superalloy structure and compare them with experimental observations. Finally, Section 6 concludes the paper.

## 2. Thermal Elastoplastic Bond-Based Peridynamic Model

**Figure 1:**
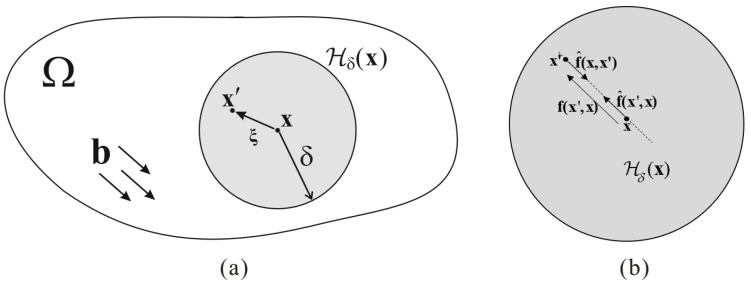
(**a**) Nonlocal continuum Ω and (**b**) partition of the interaction $f(x^{\prime },x)$ into partial interactions.

PD theory assumes that there is an interaction between non-contact material points separated by a finite distance. Therefore, in the reference configuration Ω of an object, the interaction between any point x and a point $x^{\prime }$ in its neighborhood is described by the pairwise force function f. The balance equation at point x can be written as Equation (1),

(1) $$\int_{H_{\delta }\left(x\right)}f\left(x^{\prime },x\right)dV_{x^{\prime }}+b\left(x\right)=0$$

where b is a prescribed body force density and $H_{\delta }\left(x\right)\subset \Omega$ is a circular region with x as its center and δ as its radius (see Figure 1). Thus, δ is referred to as the horizon [4].

The pairwise force function f can be defined as Equation (2)

(2) $$f\left(x^{\prime },x\right)=\hat{f}\left(x^{\prime },x\right)-\hat{f}\left(x,x^{\prime }\right),$$

where $\hat{f}\left(x^{\prime },x\right)$ is the partial interaction due to the action of point $x^{\prime }$ over point x and, correspondingly, $\hat{f}\left(x,x^{\prime }\right)$ is the partial interaction due to the action of point x over point $x^{\prime }$. By introducing plastic deformation and thermal expansion into the bond deformation calculation, the partial interaction can be written as Equation (3)

(3) $$\hat{f}\left(x^{\prime },x\right)=\frac{1}{2}c\left(x,\xi \right)\left(\eta_{\xi }\left(x^{\prime },x\right)-{\bar{\eta }}_{\xi }-a\left(x,\xi \right)\hat{T}\left(x,\xi \right)\right)e_{\xi },$$

where $\xi =x^{\prime }-x$ is the relative position vector herein referred to as “bond”, $\eta_{\xi }\left(x^{\prime },x\right)=\eta \left(x^{\prime },x\right)\cdot e_{\xi }$ denotes the projection of the bond deformation at point x over the bond, $\eta \left(x^{\prime },x\right)=u\left(x^{\prime }\right)-u\left(x\right)$, and eξ=ξξ. In addition, u is the displacement and $e_{\xi }$ is the unit direction vector of the bond. In Equation (3), $a\left(x,\xi \right)\hat{T}\left(x,\xi \right)$ represents the deformation of the bond due to thermal expansion [34], where a(x,ξ) is the coefficient of thermal expansion of the bond in the PD model. We define $\hat{T}\left(x,\xi \right)$ as the effective temperature of the bond. In other words, $\hat{T}$ is determined by T(x) and $T(x^{\prime })$, which are the temperature variations between the current temperatures and the reference temperatures of points x and $x^{\prime }$, respectively. Thus, we have Equation (4).

(4) $$\hat{T}\left(x,\xi \right)=\hat{T}\left(x^{\prime },-\xi \right).$$

Moreover, ${\bar{\eta }}_{\xi }=\bar{\eta }\cdot e_{\xi }$, where $\bar{\eta }$ is the historical plastic elongation of the bond defined by Equation (5)

(5) $$\bar{\eta }\left(0\right)=0,\;\dot{\bar{\eta }}=\left\{\begin{array}{cc} \dot{\eta } & \;\mathrm{if}\;\frac{\left|\xi +\eta -\bar{\eta }\right|-\left|\xi \right|}{\left|\xi \right|}\geq s_{Y},\\ 0 & \;\mathrm{otherwise} \end{array}\right.$$

where $s_{Y}$ is the yield stretch. At the bond level, this material is elastic-perfectly plastic. However, for the entire structure, the material model has a strain-hardening effect because not all bonds will yield at a certain moment or under a certain deformation. It is worth noting that $s_{Y}$ is an intrinsic parameter of the material, and its value is related to its engineering ultimate strength [20]. Moreover, the fracture behavior of materials can be described by bond failure [35]. It is assumed that a bond breaks irreversibly if its stretch *s* exceeds the critical value $s_{C}$ (generally, $s_{C}>s_{Y}$), where $s=\frac{\left|\xi +\eta \right|-\left|\xi \right|}{\left|\xi \right|}$.

After introducing plastic elongation at the bond level, we can calculate an average measure of non-local plastic deformation at a given point as Equation (6)

(6) $$\phi_{p}\left(x\right)=\frac{\int_{H_{\delta }\left(x\right)}\bar{s}dV_{\xi }}{\int_{H_{\delta }\left(x\right)}dV_{\xi }},$$

where $\bar{s}=\frac{|\xi +\bar{\eta }\left|-|\xi \right|}{\left|\xi \right|}$ denotes the plastic stretch of the bond.

## 3. Thermoelastic Hybrid Peridynamics and Classical Continuum Mechanics (PD-CCM) Model

Hybrid PD-CCM models retain the advantages of PD when facing discontinuities and improve calculation efficiency [10,11,12,13,14]. In this section, we adopt the Morphing method [15] to establish a thermoelastic hybrid PD-CCM model.

### 3.1. Governing Equations

**Figure 2:**
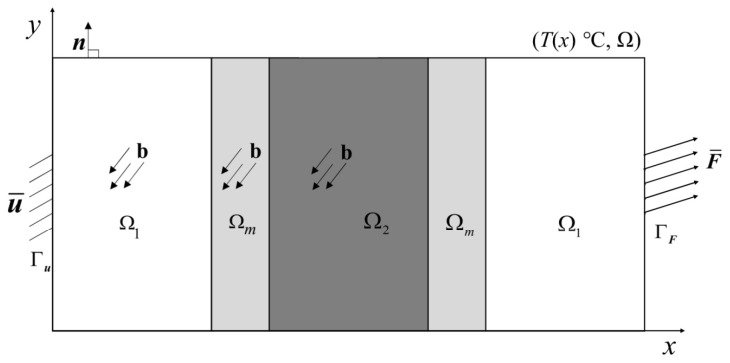
Region Ω consists of $\Omega_{1}$, $\Omega_{2}$ and $\Omega_{m}$.

We divide the whole solution region into three non-overlapping subregions, i.e., $\Omega =\Omega_{1}\cup \Omega_{2}\cup \Omega_{m}$, where $\Omega_{1}\cap \Omega_{2}=\emptyset$, $\Omega_{1}\cap \Omega_{m}=\emptyset$, and $\Omega_{2}\cap \Omega_{m}=\emptyset$. The PD model is confined to subregion $\Omega_{2}$, where plastic deformation and fracture may occur, and the CCM model is employed in subregions $\Omega_{1}$ to reduce computational costs. The two models transition through subregion $\Omega_{m}$. As shown in Figure 2, the displacement boundary condition $\bar{u}$ is applied to part $\Gamma_{u}$ of ∂Ω, and the traction load $\bar{F}$ is applied to part $\Gamma_{F}$ of ∂Ω. n is a unit vector representing the outer normal, and the entire solution region Ω is affected by the body force b and the steady-state temperature field T(x).

The Morphing method is based on a set of governing equations(Equations (7)–(13)) that are valid for any point in Ω. It defines the material properties of the two models as functions that change with position [33]. The governing equations are as follows.

- **Kinematic admissibility and compatibility**
  (7) $$\begin{array}{cccc} \varepsilon (x) & =\frac{1}{2}\left(\nabla u\left(x\right)+{\left(\nabla u\right)}^{T}\left(x\right)\right) & \; & \forall x\in \Omega \backslash \Omega_{2} \end{array}$$
  (8) $$\begin{array}{cccc} \eta_{\xi }\left(x^{\prime },x\right) & =u_{\xi }\left(x^{\prime }\right)-u_{\xi }\left(x\right) & \; & \forall x,x^{\prime }\in \Omega \backslash \Omega_{1} \end{array}$$
  (9) $$\begin{array}{cccc} u(x) & =\bar{u}\left(x\right) & \; & \forall x\in \Gamma_{u} \end{array}$$
- **Static admissibility**
  (10) $$\begin{array}{cccc} \mathrm{div}\sigma \left(x\right)+\int_{H_{\delta }\left(x\right)}f\left(x^{\prime },x\right)dV_{x^{\prime }} & =-b(x) & \; & \forall x\in \Omega \end{array}$$
  (11) $$\begin{array}{cccc} \sigma (x)\cdot n(x) & =\bar{F} & \; & \forall x\in \Gamma_{F} \end{array}$$
- **Constitutive equations**
  (12) $$\begin{array}{cccc} \sigma (x) & =E(x):\varepsilon (x)-\beta (x)T(x) & \; & \forall x\in \Omega \backslash \Omega_{2} \end{array}$$
  (13) $$\begin{array}{cccc} f\left(x^{\prime },x\right) & =\frac{1}{2}\left(c\left(x,\xi \right)+c\left(x^{\prime },\xi \right)\right)\left(\eta_{\xi }\left(x^{\prime },x\right)-{\bar{\eta }}_{\xi }\right)e_{\xi } & \; & \\ \; & -\frac{1}{2}\left(b\left(x,\xi \right)\hat{T}\left(x,\xi \right)+b\left(x^{\prime },\xi \right)\hat{T}\left(x^{\prime },-\xi \right)\right)e_{\xi } & \; & \forall x\in \Omega \backslash \Omega_{1} \end{array}$$

Here, σ and ε are the stress and strain tensors, respectively. In Equation (12), E(x) is the stiffness tensor, and β(x)=E(x):α(x) is the thermal modulus, where α(x) is the thermal expansion coefficient tensor.

For homogeneous materials, the PD micromodulus function c(x,ξ) and the thermal expansion coefficient function a(x,ξ) can be redefined as Equations (14) and (15)

(14) $$c\left(x,\xi \right)=w\left(x\right)c^{0}\left(|\xi |\right),$$

(15) $$a\left(x,\xi \right)=v\left(x\right)a^{0}\left(|\xi |\right),$$

where w(x) and v(x) are the Morphing functions and 0≤w(x),v(x)≤1,∀x∈Ω. $c^{0}\left(|\xi |\right)$ and $a^{0}\left(|\xi |\right)$ are the PD micromodulus and thermal expansion coefficient, respectively. We define the PD thermal modulus as $b^{0}\left(|\xi |\right)=c^{0}\left(|\xi |\right)a^{0}\left(|\xi |\right)$. Then, the pairwise force function f can be rewritten as in Equation (13), where b(x,ξ) can be defined by Equation (16)

(16) $$b\left(x,\xi \right)=w\left(x\right)v\left(x\right)b^{0}\left(|\xi |\right).$$

### 3.2. Stiffness/Thermal Modulus Constraint Equations

The governing equations of the hybrid model were presented in Section 3.1. Functions w(x), v(x) and E(x), β(x) jointly guide the transition between the CCM and PD models. w(x) and v(x) determine the division of the hybrid model; thus, how we define E(x) and β(x) is key to successfully couple both models together. In this subsection, we derive the constraint functions for E(x) and β(x) based on the point-by-point equivalency of the energy density.

Plastic deformation is confined to the pure peridynamic region, that is, ${\bar{\eta }}_{\xi }\equiv 0$ outside this region. Thus, the constitutive equation defined in Equation (13) can be simplified to an elastic version. Let us consider a subregion $\Omega_{0}=\left\{x\in \Omega :H_{\delta }\left(x\right)\subset \Omega \right\}$, such that we still have a complete integral region when the PD model is used near the boundary. We can derive the following equations based on a previous study [33]. The following assertions hold true for any point $x\in \Omega_{0}$:

- If and only if $E\left(x\right)=E^{0}$, $\beta \left(x\right)=\beta^{0}$ and $w\left(x^{\prime }\right),v\left(x^{\prime }\right)=0$, $\forall x^{\prime }\in H_{\delta }\left(x\right)$, the model is restricted to the pure CCM model at point x. Then the elastic energy density at point x can be written as Equation (17)
  (17) $$W\left(x\right)=\frac{1}{2}\varepsilon \left(x\right):E^{0}:\varepsilon \left(x\right)-\varepsilon \left(x\right):\beta^{0}T\left(x\right)+W^{0}\left(T\right).$$
- If and only if E(x)=0, β(x)=0 and $w\left(x^{\prime }\right),v\left(x^{\prime }\right)=1$, $\forall x^{\prime }\in H_{\delta }\left(x\right)$, the model is restricted to the pure PD model at point x. Considering Equation (4), the elastic energy density at point x can be written as Equation (18)
  (18) $$W\left(x\right)=\frac{1}{4}\int_{H_{\delta }\left(x\right)}c^{0}\left(|\xi |\right)\eta_{\xi }^{2}\left(x^{\prime },x\right)dV_{x^{\prime }}-\frac{1}{2}\int_{H_{\delta }\left(x\right)}b^{0}\left(|\xi |\right)\hat{T}\left(x,\xi \right)\eta_{\xi }\left(x^{\prime },x\right)dV_{x^{\prime }}+W^{0}\left(T\right).$$
- If and only if E(x)≠0, β(x)≠0 and $\exists x^{\prime }\in H_{\delta }\left(x\right)$ such that 0<w(x)<1,0<v(x)≤1, the hybrid model must be used at point x. Considering Equation (4), the elastic energy density at point x can be written as Equation (19)
  (19) $$\begin{array}{c} W\left(x\right)=\frac{1}{2}\varepsilon \left(x\right):E\left(x\right):\varepsilon \left(x\right)+\frac{1}{4}\int_{H_{\delta }\left(x\right)}c^{0}\left(|\xi |\right)\frac{w\left(x^{\prime }\right)+w\left(x\right)}{2}\eta_{\xi }^{2}\left(x^{\prime },x\right)dV_{x^{\prime }}+W^{0}\left(T\right)\\ -\varepsilon \left(x\right):\beta \left(x\right)T\left(x\right)-\frac{1}{2}\int_{H_{\delta }\left(x\right)}b^{0}\left(|\xi |\right)\frac{w\left(x^{\prime }\right)v\left(x^{\prime }\right)+w\left(x\right)v\left(x\right)}{2}\hat{T}\left(x,\xi \right)\eta_{\xi }\left(x^{\prime },x\right)dV_{x^{\prime }}. \end{array}$$

**Remark 1.**  *$W^{0}\left(T\right)$ corresponds to the $T^{2}$ term in the elastic energy density equations.*

For homogeneous materials, the elastic energy density at any point should be independent of the definition of the Morphing functions w(x) and v(x) and only depend on the displacement and temperature field. This implies that the elastic energy densities calculated at the same point with different expressions are equivalent to each other; thus, the corresponding terms of force and temperature must be equal. Therefore, the terms in Equation (17) are equal to the corresponding ones in Equation (18), then we have Equations (20) and (21)

(20) $$\begin{array}{cc} \frac{1}{2}\varepsilon \left(x\right):E^{0}:\varepsilon \left(x\right) & =\frac{1}{4}\int_{H_{\delta }\left(x\right)}c^{0}\left(|\xi |\right)\eta_{\xi }^{2}\left(x^{\prime },x\right)dV_{x^{\prime }}, \end{array}$$

(21) $$\begin{array}{cc} \varepsilon \left(x\right):\beta^{0}T\left(x\right) & =\frac{1}{2}\int_{H_{\delta }\left(x\right)}b^{0}\left(|\xi |\right)\hat{T}\left(x,\xi \right)\eta_{\xi }\left(x^{\prime },x\right)dV_{x^{\prime }}. \end{array}$$

Furthermore, we assumed that both the temperature and elastic deformation in a small neighborhood around point x are uniform; thus, we have Equations (22) and (23)

(22) $$\varepsilon \left(x^{\prime }\right)≃\varepsilon \left(x\right)=\bar{\varepsilon }\;\mathrm{and}\;\eta_{\xi }\left(x^{\prime },x\right)=\frac{\xi \cdot \bar{\varepsilon }\cdot \xi }{\left|\xi \right|},\forall x^{\prime }\in H_{\delta }\left(x\right),$$

(23) $$\hat{T}\left(x,\xi \right)≃T\left(x\right)=\bar{T},\forall x^{\prime }\in H_{\delta }\left(x\right).$$

Thus, Equations (20) and (21) lead to Equations (24) and (25)

(24) $$\begin{array}{cc} E^{0} & =\frac{1}{2}\int_{H_{\delta }\left(x\right)}c^{0}\left(|\xi |\right)\frac{\xi ⊗\xi ⊗\xi ⊗\xi }{{\left|\xi \right|}^{2}}dVx^{\prime }, \end{array}$$

(25) $$\begin{array}{cc} \beta^{0} & =\frac{1}{2}\int_{H_{\delta }\left(x\right)}b^{0}\left(|\xi |\right)\frac{\xi ⊗\xi }{\left|\xi \right|}dVx^{\prime }. \end{array}$$

Similarly, the terms of Equation (17) are equal to the corresponding ones in Equation (19). By considering Equations (22)–(25) we get Equations (26) and (27)

(26) $$\begin{array}{cc} E(x) & =E^{0}-\frac{1}{2}\int_{H_{\delta }\left(x\right)}c^{0}\left(|\xi |\right)\frac{w\left(x^{\prime }\right)+w\left(x\right)}{2}\frac{\xi ⊗\xi ⊗\xi ⊗\xi }{{\left|\xi \right|}^{2}}dVx^{\prime }, \end{array}$$

(27) $$\begin{array}{cc} \beta (x) & =\beta^{0}-\frac{1}{2}\int_{H_{\delta }\left(x\right)}b^{0}\left(|\xi |\right)\frac{w\left(x^{\prime }\right)v\left(x^{\prime }\right)+w\left(x\right)v\left(x\right)}{2}\frac{\xi ⊗\xi }{\left|\xi \right|}dVx^{\prime }. \end{array}$$

In summary, we have established a thermoelastic hybrid PD-CCM model in Equations (7)–(16), (26) and (27). The partitioning of the hybrid model is determined by the Morphing functions w(x) and v(x), and the definitions of w(x) and v(x) are independent of each other.

## 4. Plastic-Fracture Calculations

In this study, the constraint equations of the hybrid model ensure that PD is applied only in the critical region where plastic deformation and cracks may occur. The plastic bond constitutive equation defined in Equation (3) is used to perform iterative calculations.

**Figure 3:**
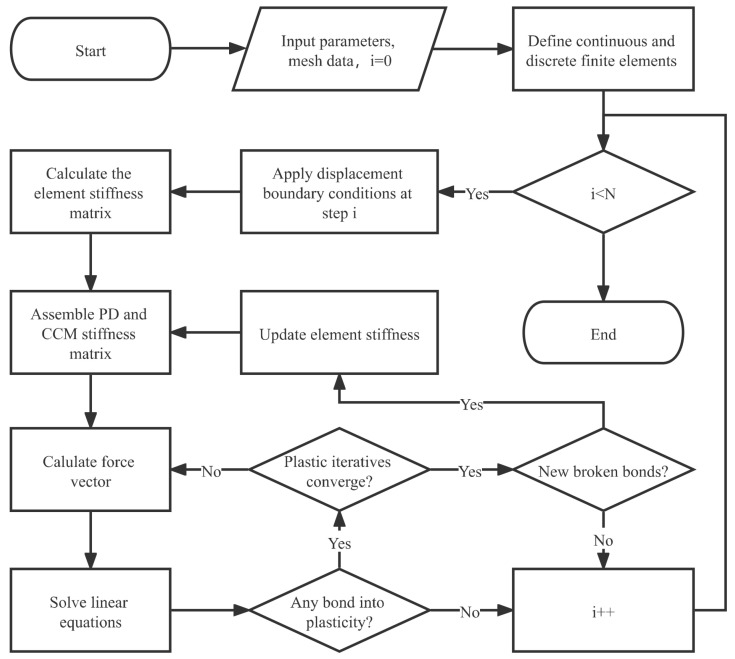
Flowchart of the iterative algorithm of the peridynamic plastic-fracture model.

Figure 3 shows a flowchart of the implicit iterative algorithm used for the plastic-fracture simulation. In this algorithm, the coupling and the CCM zones are discretized using continuous finite elements, whereas the pure PD zone is discretized using discrete finite elements, i.e., nodes are not shared between different elements. Discrete elements allow the cracks to grow freely along their boundaries. In fact, we can also discrete the PD zone by continuous elements, then cracks can pass through the element and manifest as damage. The discrete strategy may affect the crack propagation path, but the crack propagation simulated by these two discrete strategies is consistent when appropriate mesh fineness is used. The two discrete strategies are discussed in the reference [36].

The boundary conditions are gradually imposed in N increments. At each incremental step, the elongation of each bond is calculated based on the displacement field obtained by solving the corresponding linear equations. If any bond yields, the residual plastic force must be calculated to update the force vector on the right side of the equations. This step is repeated until the iterative solution of the system of linear equations converges.

After the plastic iterative calculation converges, if any bonds break at the current step, we must update the stiffness matrix by removing the contribution of these bonds. Change in the stiffness matrix will inevitably alter the calculated displacement field; therefore, we must recalculate the residual plastic force and solve the equations until the plastic iteration converges and no bonds break at the current step. Subsequently, the step counter is incremented and calculations are repeated until the last load step is completed.

## 5. Numerical Examples

In this section, we validate the proposed model by presenting two numerical examples. The first is a two-dimensional simplified model of an actual experiment involving a GH4099 superalloy structure in high-temperature environments. We compare the simulated fracture modes and force–displacement curves with the experimental results to assess the ability of the proposed model for simulating the failure of ductile materials. The second numerical example involves a four-point bending beam under uniform and non-uniform temperature fields to investigate the effects of temperature field and plastic deformation on crack propagation. The numerical examples are calculated by the home-made program based on C++, which is designed in parallel using OpenMP. All calculations were performed on the 12th Gen Intel(R) Core(TM) i7-12700F processor.

It is important to note that the zone in which PD is implemented in the numerical simulations is empirically determined. In previous studies, Wang, Han and Lubineau proposed adaptive coupling modeling techniques based on broken-bond drive [31] and strength drive [32], to update the PD zone and remesh so as to track the crack propagation path. However, For the numerical examples presented in this study, it is feasible to preset a fixed PD zone that can cover the crack path. Therefore, the adaptive coupling modeling and remesh techniques are not considered in the plastic fracture simulations in this study.

### 5.1. Example 1: GH4099 Superalloy Structure

**Figure 4:**
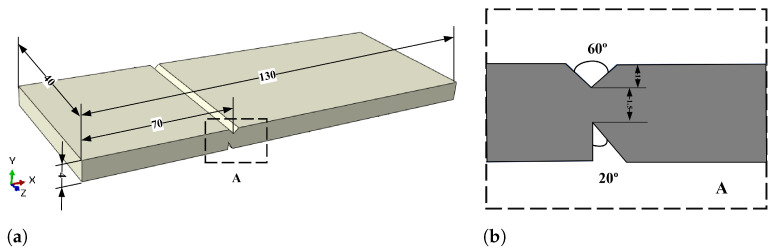
(**a**) Schematic diagram of the experimental specimen geometry (unit: mm) and (**b**) groove details.

**Figure 5:**
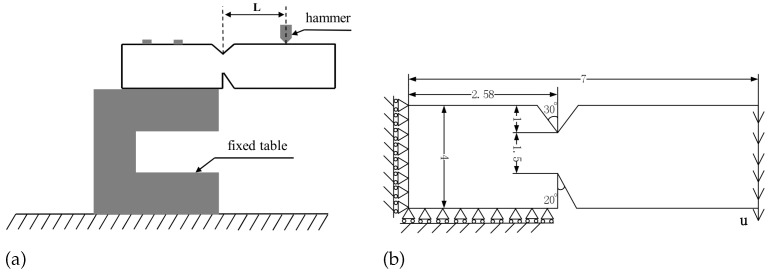
(**a**) Experimental setup and (**b**) simplified numerical model. (unit: mm).

The geometry of the experimental GH4099 superalloy specimen is presented in Figure 4. As shown in Figure 4b, the top of the specimen is preset with a $60^{{}^{\circ}}$ V-shaped groove with a depth of 1 mm, whereas the bottom was preset with a $20^{{}^{\circ}}$ angle weakening groove with a depth of 1.5 mm. The experimental setup is shown in Figure 5a. The distance between the loading hammer and the weakening groove is L=24.5 mm. First, the structures are heated to the target temperatures (800 °C and 900 ℃ respectively) at the rate of 30 ℃ per minute in a high-temperature chamber, and holding temperature for 5 min. Then the hammer as shown in Figure 5a was vertically loaded downward at the speed of 0.5 mm per minute. The measurement tolerance of the high-temperature chamber is ±2 ℃. And the measurement resolution of hammer loading force is 0.001N.

The focus of this simulation was on the crack mode. The cracking position of the structure is far away from the load application region, and its deformation is only related to the resultant force and moment of the load. According to the Saint–Venant principle, the specific distribution of the load affects only the stress distribution around the load application region. The error caused by this approximation is confined near the loading boundary. Therefore, considering the computational efficiency requirements, we scaled the length dimension according to the equivalent of the resultant moment at the groove position. On the other hand, because the width-to-thickness ratio of the experimental specimen is 10:1, we adopted the plane strain assumption to simplify the three-dimensional structure to a two-dimensional numerical specimen. The dimensions of the numerical specimen and the boundary conditions are shown in Figure 5b.

**Figure 6:**
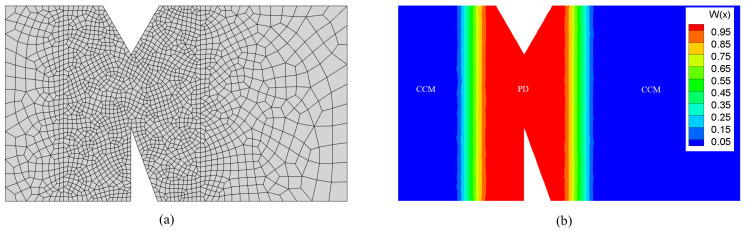
(**a**) Grid and (**b**) value distribution of the Morphing function. (for interpretation of the references to color in this figure, the reader is referred to the web version of this paper).

A grid of the geometric model is shown in Figure 6a. We selected a small mesh fineness in PD zone to ensure the precision of crack simulations, while a larger mesh fineness was selected in CCM zone to reduce the calculation consumption. The study on mesh convergence of PD-CCM coupling model based on the Morphing method can be found in the reference [16]. The model was discretized using four-node quadrilateral elements into 2093 nodes and 2009 elements. The grid size was 0.1 mm in the region delimited by y∈[0,4],x∈[1.2,4] and uniformly transitioned outside of this region from 0.1 mm along the *x* direction to 0.4 mm at the two boundaries. Figure 6b shows the value distribution of the Morphing function w(x). From Equations (26) and (27), it can be seen that the model partitions are determined by the values of w(x) and w(x)v(x). We preset v(x)=1 for the entire body Ω, that is, the model partition was only affected by the value distribution of w(x). Thus, in Figure 6b, the red region corresponds to the pure PD model (w(x)=1), the blue region corresponds to the pure CCM model (w(x)=0), and the transition region corresponds to the coupled model (0<w(x)<1).

**Figure 7:**
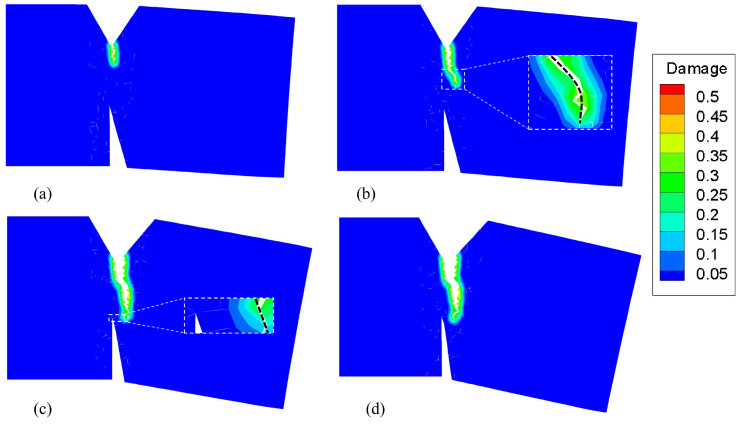
Damage contour and crack propagation path of a superalloy structure at 800 ℃ at the (**a**) 15th step, (**b**) 20th step, (**c**) 40th step, and (**d**) 50th step. (for interpretation of the references to color in this figure, the reader is referred to the web version of this paper).

Because we used a bond-based PD model, Poisson’s ratio for plane strain problems was fixed at 1/4 [4,37]. The horizon δ of the PD model was set to 0.3 mm and the displacement increment was $\Delta \bar{u}=$ 0.018 mm. The material parameters are shown in Table 1, at 800 ℃, the Young’s modulus of the GH4099 superalloy is 147 GPa, and its thermal expansion coefficient is $15.1\times 10^{-6}\;{{}^{\circ}C}^{-1}$. The yield ($s_{Y}$) and critical ($s_{C}$) stretch were set to 0.04 and 0.1 respectively. Figure 7a shows the damage contour at the 15th loading step. Owing to the ductility of the superalloy at 800 ℃, the initial crack propagation direction was affected by the V-groove at the top of the structure and had a certain angle with the vertical direction, which was different from that of a brittle fracture. As loading progressed, the crack continued to propagate along the initial direction. By the 20th loading step (see Figure 7b), the crack started to deflect toward the bottom weakening groove. By the 40th loading step (see Figure 7c), the crack had propagated close to the tip of the weakening groove, and the crack propagation direction became parallel to the hypotenuse of the weakening groove. In this state, It is difficult for the structure to fracture completely. Thus, after the 50th loading step (see Figure 7d), the crack did not continue to grow as the loading increased.

**Figure 8:**
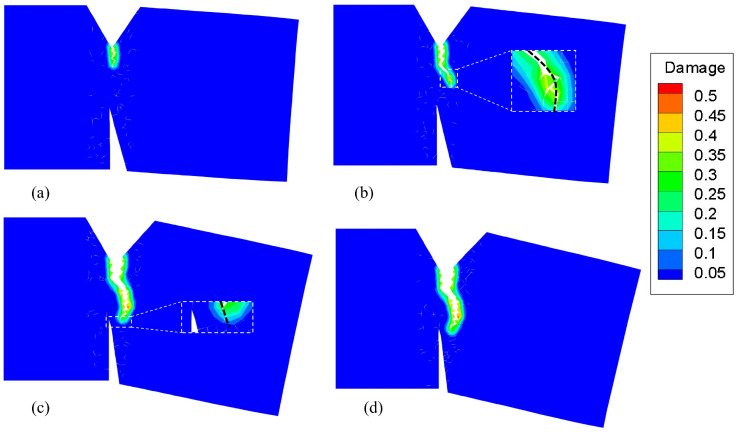
Damage contour and crack propagation path of a superalloy structure at 900 ℃ at the (**a**) 16th step, (**b**) 24th step, (**c**) 48th step, and (**d**) 54th step. (for interpretation of the references to color in this figure caption, the reader is referred to the web version of this paper).

**Figure 9:**
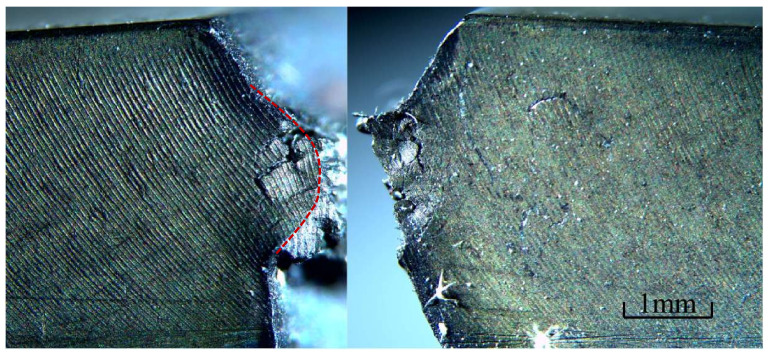
Experimental observations of the final fracture pattern at 900 ℃.

At 900 ℃, the Young’s modulus of the GH4099 superalloy is 121 GPa and its thermal expansion coefficient is $15.3\times 10^{-6}\;{{}^{\circ}C}^{-1}$. The yield ($s_{Y}$) and critical ($s_{C}$) stretch were set to 0.04 and 0.12, respectively. The simulation results obtained in a 900 ℃ environment are shown in Figure 8. The entire fracture process is similar to that at 800 ℃. The direction of crack propagation changed from the 16th step to the 24th step, as shown in Figure 8a,b. Subsequently, the crack propagated close to the tip of the weakening groove, and the path became parallel to the hypotenuse of the weakening groove by the 48th step. After the 54th loading step, the crack stoped growing. However, the crack propagation direction deflected in a more pronounced manner compared with the results obtained at 800 ℃. Figure 9 shows a photomicrograph of the GH4099 superalloy structure at 900 ℃. An arc-shaped crack path consistent with the simulated crack path can be observed in the figure.

**Figure 10:**
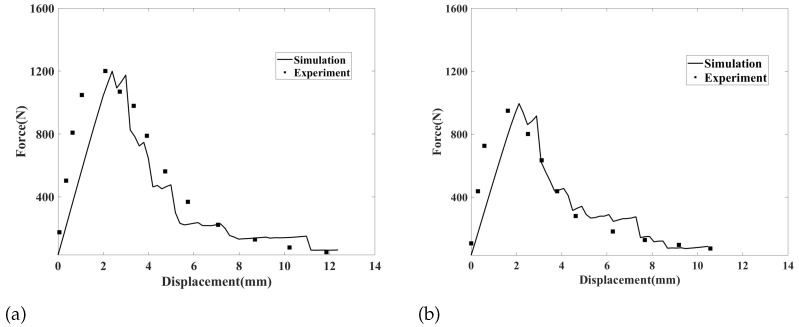
Simulated and experimental force-displacement curves at (**a**) 800 ℃ and (**b**) 900 ℃.

To further verify the effectiveness of the proposed model for simulating ductile fractures at high temperatures, we compared the force-displacement curves obtained from the simulations and experiments at 800 ℃ and 900 ℃. The results are shown in Figure 10. Unlike in brittle fractures, force did not quickly decrease to zero after reaching its peak value. The simulation results were essentially consentient with the experimental results, and the error of simulated peak forces at 800 ℃ and 900 ℃ relative to experimental results is 0.9% and 4.9%, respectively. In both the simulations and experiments, at the end of the curve, the force did not decrease with an increase in displacement but instead remained constant. This indicates that, in the final stage of the failure process of superalloy structures, the structure does not fracture further as loading progresses. This coincides with the simulated final crack mode. By comparing the force-displacement curves obtained at 800 ℃ and 900 ℃, we can see that the peak force at 900 ℃ was significantly lower than that at 800 ℃ owing to the softening effect of the high-temperature environment on the alloy. Additionally, the displacements of both crack initiation and final break decreased, indicating that superalloy structures become more prone to failure as temperature increases.

### 5.2. Example 2: Four-Point Bending Beam

**Figure 11:**
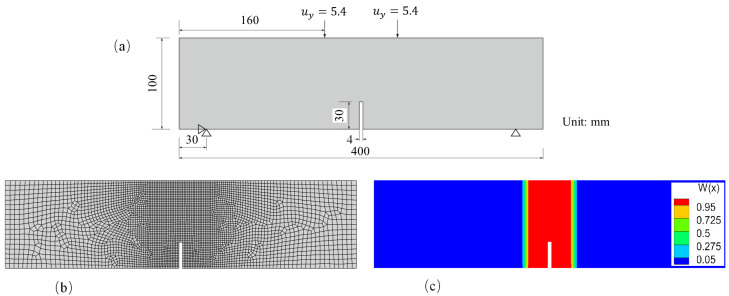
Schematic diagram of the four-point bending beam: (**a**) geometry and boundary conditions, (**b**) grid and (**c**) value distribution of the Morphing function. (for interpretation of the references to color in this figure, the reader is referred to the web version of this paper).

**Figure 12:**
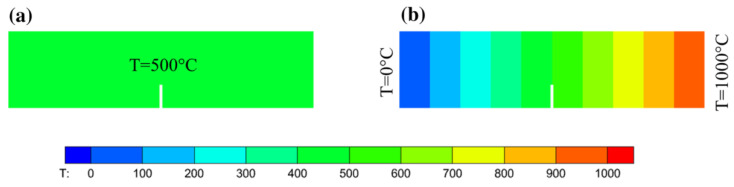
Two different temperature conditions used for the numerical simulations of the four-point bending beam: (**a**) uniform temperature field, (**b**) non-uniform temperature field. (for interpretation of the references to color in this figure, the reader is referred to the web version of this paper).

This example illustrates the effects of the temperature field and plastic deformation on the crack propagation paths. The geometry and boundary conditions for this numerical example are shown in Figure 11a. The grid used in this example is shown in Figure 11b. The grid size in the PD region was set to 2mm. Figure 11c shows the value distribution of the Morphing function w(x), which indicates the scope of the different models. The fracture process of the four-point bending beam was simulated under two different temperature conditions: a uniform temperature field of 500 ℃ (Figure 12a) and a non-uniform temperature field transitioning from 0 ℃ to 1000 ℃ (Figure 12b). This setting ensures that the specimen was always at 500 ℃ in the vertical direction of the beam notch.

To consider the effects of plastic deformation and temperature on the crack path of the four-point bending beam, we conducted four numerical tests with the same Young’s modulus and thermal expansion coefficient. The parameter settings for the four numerical tests are listed in Table 2. In all tests, the horizon δ was set to three times the grid size of the PD region. This example assumes a plane-stress condition; therefore, Poisson’s ratio was fixed at 1/3.

**Figure 13:**
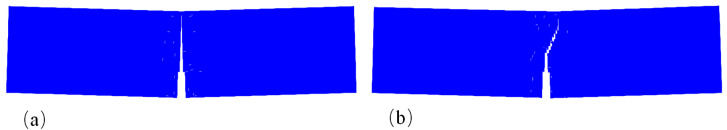
Final crack paths of (**a**) case 1 and (**b**) case 3.

**Figure 14:**
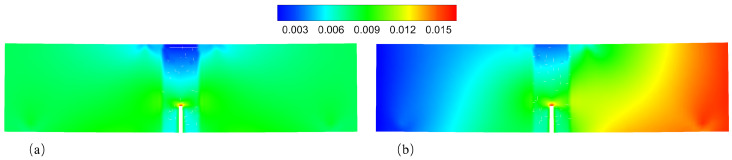
Strain $\varepsilon_{xx}$ contours before the crack initiation of (**a**) case 1 and (**b**) case 3.

First, we compared the crack paths of the four-point bending beam under different temperature conditions. Figure 13 shows the final crack paths for cases 1 and 3. These two cases had the same parameter settings except for temperature, but nonetheless exhibited different crack patterns. Under the uniform temperature field (see Figure 13a), the crack propagated along the vertical direction of the beam notch. In contrast, it propagated along a different path under the non-uniform temperature field (see Figure 13b). We presented the strain contours before crack initiation of both cases 1 and 3. Figure 14a shows the strain of $\varepsilon_{xx}$ under the uniform temperature condition, in which the beam has a symmetrical strain field. It is obvious that the crack will propagate vertically. In the case of non-uniform temperature condition, the transverse temperature gradient leads to the asymmetric strain field (see Figure 14b), and the value of strain $\varepsilon_{xx}$ in the right half of the beam is significantly higher than that in the left half. Therefore, the crack will deviate from the center line to the right.

**Figure 15:**
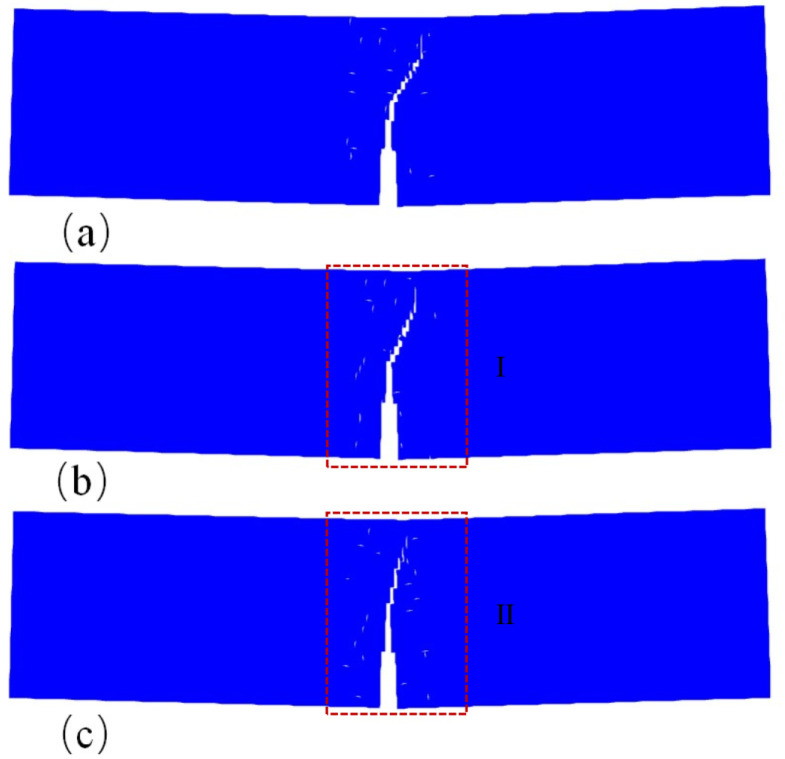
Crack paths for the three cases under a non-uniform temperature field: (**a**) case 2, (**b**) case 3, and (**c**) case 4.

In cases 2, 3, and 4, the numerical simulations were performed under the non-uniform temperature field, but plastic deformation was only allowed in cases 3 and 4. Thus, in case 2, bonds broke immediately after their deformation reached the elastic limit (which is considered brittle fracture). In cases 3 and 4, an admissible plastic elongation of $\bar{s}=0.005$ and $\bar{s}=0.01$ was introduced after the deformation of a single bond reaches the elastic limit, respectively. The elastic limit of the bond was the same in all three cases. Figure 15 shows the final crack paths of the four-point bending beam for cases 2, 3 and 4. By comparing the crack paths of these three cases, it can be seen that the angle of the crack path in case 2 deviated the most from the vertical direction under the non-uniform temperature field (see Figure 15a). In addition, the deflection angle of the crack path decreased with an increase in the admissible plastic deformation of the bond.

**Figure 16:**
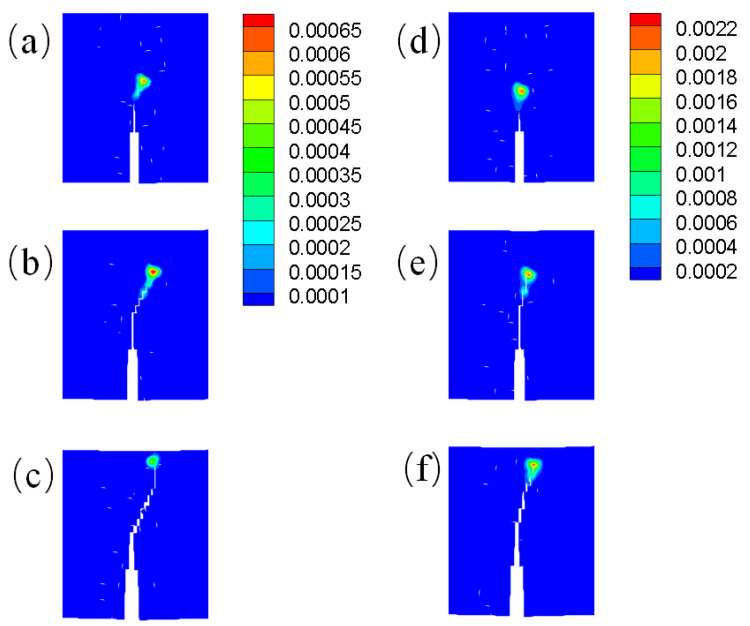
Nonlocal plastic deformation contours: (**a**–**c**) correspond to the 10th, 15th, and 25th loading steps for region I in Figure 15, respectively, (**d**–**f**) correspond to the 10th, 15th, and 25th loading steps for region II in Figure 15, respectively. (For interpretation of the references to color in this figure caption, the reader is referred to the web version of this paper).

We also analyzed the plastic deformation zone of the crack tip during crack propagation in cases 3 and 4. In Figure 16, subfigures (a), (b) and (c) show the plastic deformation zones around the crack tip at the 10th, 15th and 25th loading steps, respectively, corresponding to region I in Figure 15. On the other hand, subfigures (d), (e), and (f) show the plastic deformation zones around the crack tip at the 10th, 15th and 25th loading steps, corresponding to region II in Figure 15. Color indicates the value of the non-local plastic deformation calculated using Equation (6). Compared with case 3, the crack tip in case 4 exhibited a greater nonlocal plastic deformation during the crack propagation. This greater nonlocal plastic deformation around the crack tip weakens the influence of the temperature gradient on the crack path. Therefore, in case 4, the angle at which the crack path deviated from the vertical directionwas smaller than that in case 3.

## 6. Conclusions

We propose a PD modeling approach to simulate the plastic deformation and fracture of structures in a thermomechanical coupling field. The proposed model can qualitatively capture the ductile fracture mode of structures. We simulated the plastic-fracture process of a GH4099 superalloy structure at 800 ℃ and 900 ℃. The simulation results showed that the proposed model predicts an arc-shaped crack path, which is consistent with experimental observations. The simulated crack path and force--displacement curves were essentially consentient with the experimental results, demonstrating the effectiveness of the proposed model for simulating ductile fractures at high temperatures. We also conducted numerical tests on a four-point bending beam under uniform and non-uniform temperature fields, and discussed the effects of temperature and plastic deformation on the crack path. The results show that the plastic deformation zone around the crack tip weakened the influence of temperature on crack deflection.

The proposed model has the advantage of PD in dealing with fracture problems, and the coupling with CCM improves the efficiency and applicability of the model. The PD-CCM coupled model has great potential in the simulation of plastic fracture in thermomechanical coupling field. However, compared with the CCM model, PD constitutive equations still lack experimental verification and verification. Therefore, further research can focus on the experimental verification and verification of PD constitutive equations.

## Figures and Tables

**Table 1:** Material and numerical parameters of the GH4099 Superalloy Structure.

Temperature	Young’s Modulus (GPa)	Coefficient of Thermal Expansion ($10^{-6}\;{{}^{\circ}C}^{-1}$)	$s_{Y}$	$s_{C}$	Increments
800 ℃	147	15.1	0.04	0.1	50
900 ℃	121	15.3	0.04	0.12	50

**Table 2:** Material and numerical parameters of four-point bending beam.

Cases	Young’s Modulus (GPa)	Coefficient of Thermal Expansion ($10^{-6}\;{{}^{\circ}C}^{-1}$)	$s_{Y}$	$s_{C}$	Temperature Conditions	Increments
case 1	200	15.6	0.02	0.025	Figure 12a	30
case 2	200	15.6	∖	0.02	Figure 12b	30
case 3	200	15.6	0.02	0.025	Figure 12b	30
case 4	200	15.6	0.02	0.03	Figure 12b	30

## Data Availability

Data sharing is not applicable to this article.
